# Shifting osteogenesis in vascular calcification

**DOI:** 10.1172/jci.insight.143023

**Published:** 2021-05-24

**Authors:** Jiayi Yao, Xiuju Wu, Xiaojing Qiao, Daoqin Zhang, Li Zhang, Jocelyn A. Ma, Xinjiang Cai, Kristina I. Boström, Yucheng Yao

**Affiliations:** 1 Division of Cardiology, Department of Medicine, David Geffen School of Medicine at University of California, Los Angeles (UCLA), Los Angeles, California, USA.; 2 Molecular Biology Institute, UCLA, Los Angeles, California, USA.

**Keywords:** Vascular Biology, Cardiovascular disease

## Abstract

Transitions between cell fates commonly occur in development and disease. However, reversing an unwanted cell transition in order to treat disease remains an unexplored area. Here, we report a successful process of guiding ill-fated transitions toward normalization in vascular calcification. Vascular calcification is a severe complication that increases the all-cause mortality of cardiovascular disease but lacks medical therapy. The vascular endothelium is a contributor of osteoprogenitor cells to vascular calcification through endothelial-mesenchymal transitions, in which endothelial cells (ECs) gain plasticity and the ability to differentiate into osteoblast-like cells. We created a high-throughput screening and identified SB216763, an inhibitor of glycogen synthase kinase 3 (GSK3), as an inducer of osteoblastic-endothelial transition. We demonstrated that SB216763 limited osteogenic differentiation in ECs at an early stage of vascular calcification. Lineage tracing showed that SB216763 redirected osteoblast-like cells to the endothelial lineage and reduced late-stage calcification. We also found that deletion of GSK3β in osteoblasts recapitulated osteoblastic-endothelial transition and reduced vascular calcification. Overall, inhibition of GSK3β promoted the transition of cells with osteoblastic characteristics to endothelial differentiation, thereby ameliorating vascular calcification.

## Introduction

Transitions between cell lineages contribute to the normal developmental process, whereas misguided transitions can initiate pathological processes (1–4). One such medical condition is vascular calcification, which significantly increases morbidity and mortality in diabetic patients; worsens atherosclerosis; and is associated with an increased risk of congestive heart failure, myocardial infarction, and systemic hypertension (5–8). Vascular calcification is also a significant problem in chronic kidney disease and frequently complicates vascular procedures and surgeries (9, 10). Moreover, vascular calcification has been shown to be a strong predictor of coronary heart disease, stroke, and diabetes-related amputation (11). There is currently no medical treatment that limits or reverses vascular calcification.

Vascular calcification is recognized as a common and active process of ectopic bone formation. In this process, dysregulated systemic and local factors cause vascular cells to take on osteogenic characteristics (12–15). The vascular endothelium has been shown to be a significant contributor to vascular calcification. Besides being a source of osteoinductive factors responding to hyperglycemia, oscillatory shear stress, or hyperlipidemia (12, 16, 17), the endothelium also contributes cells directly to the calcifying process (1, 12, 13, 16–20). Osteoblast-like cells derived from endothelial cells (ECs) can be detected in calcified arterial media, atherosclerotic lesions, tumor vessels, diabetic vasculopathy, and other medical conditions (1, 12–14, 16–18, 20). In these conditions, the vascular endothelium undergoes full or partial endothelial-mesenchymal transitions in order to generate calcifying cells. During the transition, the endothelial characteristics diminish while mesenchymal characteristics emerge, allowing the ECs to gain plasticity that is required to divert to osteogenic differentiation (1, 12, 13, 18–20). However, it is unknown whether it is possible to reverse the transition by shifting osteoblast-like cells to endothelial differentiation and whether this would affect vascular calcification.

In this study, we created a high-throughput screening and identified a glycogen synthase kinase 3 (GSK3) inhibitor (SB216763) that induced osteoblastic-endothelial transition. We demonstrated that SB216763 limited osteogenic characteristics in the endothelium at an early stage of vascular calcification. Lineage tracing further showed that SB216763 redirected osteoblast-like cells to endothelial differentiation and reduced late-stage calcification. Moreover, we found that limiting GSK3β resulted in osteoblastic-endothelial transition, and gene deletion of GSK3β reduced vascular calcification.

## Results

### High-throughput screening shows that the GSK3 inhibitor SB216763 promotes osteoblastic-endothelial transition.

**Figure 1:**
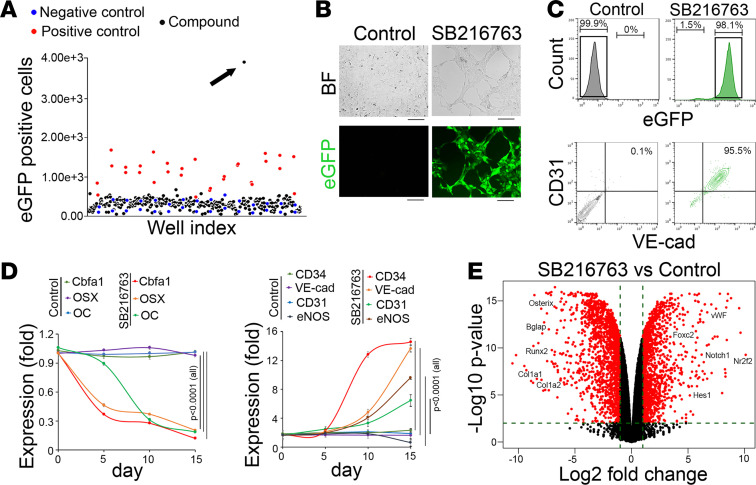
High-throughput screening identifies SB216763 as an inducer of osteoblasts to endothelial transition. (**A**) High-throughput screening showed that SB216763 induced EGFP expression in Flk1-EGFP osteoblasts as indicated by the black arrow. (**B**) Morphology of Flk1-EGFP osteoblasts after SB216763 treatment (*n* = 9). Scale bar: 50 μm. BF, bright-field. (**C**) FACS analysis of SB216763-treated Flk1-EGFP osteoblasts (top). The EGFP-positive cells (boxed) were further examined by using anti-CD31 and anti–VE-cadherin (VE-cad) antibodies (bottom) (*n* = 8). Nontreatment was used as control. (**D**) Time-course expression of the osteogenic markers Cbfa1, osterix (OSX), and osteocalcin (OC) and the endothelial markers CD34, VE-cadherin, CD31, and eNOS in SB216763-treated osteoblasts (*n* = 8). (**E**) A volcano plot of the expression profile of SB216763-treated osteoblasts versus controls (*n* = 3). Mouse pulmonary endothelial cells (ECs) were isolated by FACS and used as controls. **D** was analyzed for statistical significance by ANOVA with post hoc Tukey’s analysis. Data are shown as mean ± SD. Experiments in **B**–**D** were repeated at least 3 times.

We created a high-throughput screen by modifying the mouse osteoblast line MC3T3. Fetal liver kinase 1 (Flk1) is a well-established endothelial marker (21). We integrated the Flk1 promoter–driven EGFP into the osteoblasts, where it was undetectable at baseline (Figure 1, A and B). Induction of EGFP in the osteoblasts would indicate that the cells undergo osteoblastic-endothelial transition. We then screened several libraries of small molecules, including an FDA-approved drug library, a UCLA in-house collection, and a custom set of compounds. These libraries contained more than 22,000 small molecules ranging from natural products to synthesized compounds. After 14 days of treatment, the high-throughput screen identified the GSK3 inhibitor SB216763 as a strong activator of EGFP expression (Figure 1, A and B). After treating the cells with 10 μM SB216763, flow cytometric analysis showed that more than 98% of cells expressed EGFP, and 95% of EGFP-positive cells expressed the endothelial markers CD31 and VE-cadherin (Figure 1C). Using different doses of SB216763 (1–10 μM), we further showed that the reduction of the osteogenic markers Cbfa1, osterix, and osteocalcin and the induction of the endothelial markers CD34, VE-cadherin, CD31, and endothelial NOS (eNOS) occurred in a dose-dependent manner (Supplemental Figure 1A; supplemental material available online with this article; https://doi.org/10.1172/jci.insight.143023DS1). We treated the osteoblasts with 10 μM SB216763 and examined the time-course expression of the osteogenic and endothelial markers. The results showed that the reduction of the osteogenic markers preceded the induction of the endothelial markers (Figure 1D), suggesting that the osteoblasts lost at least part of their lineage characteristics before expressing endothelial markers. We confirmed this finding in the human osteoblast line hFOB 1.19, where the expression pattern of lineage markers was similar to that of the mouse osteoblasts (Supplemental Figure 1, B and C), suggesting that osteoblastic to endothelial transition through GSK3 inhibition is similar in human and mouse cells. To determine whether an intermediate mesenchymal stage or emerging stemness is required for SB216763 to induce the osteoblastic-endothelial transition, we examined several mesenchymal and stem cell markers but found no induction after SB216763 treatment (Supplemental Figure 2A). We further determined the specificity of endothelial differentiation by closely examining other vascular lineage markers, including smooth muscle cells (SMCs), pericytes, and fibroblast markers, using real-time PCR. In addition, we examined nonvascular lineages, including the cardiac, neuronal, hepatic, pulmonary, renal, hematopoietic, and adipogenic lineages. No changes were found in these markers (Supplemental Figure 2A). Because GSK3 is important in glucose metabolism, we determined the expression of genes involved in glucose metabolism in the SB216763-treated osteoblasts, including casein kinase 2, glucose transporter 4, and insulin receptor substrates 1 and 2. No changes were detected in these genes either (Supplemental Figure 2B).

We next examined the differential expression profiles of SB216763-treated osteoblasts using RNA-Seq, which showed that SB216763 altered the transcript profile of the cells. It suppressed osteogenic markers while enhancing endothelial markers (Figure 1E), thereby increasing the similarities between the osteoblasts treated with SB216763 and the ECs (Supplemental Figure 3A). Using a 4-fold differential threshold level, we found that the expression of 827 genes decreased in the SB216763-treated osteoblasts. These overlapped with 1357 genes with high expression in the osteoblasts as compared with ECs. We identified a 307-gene cohort in both analyses. Gene Ontology (GO) termed these genes enriched for the signal pathway of osteoblastic differentiation and bone development (Supplemental Figure 3B). Similarly, we found 519 genes with increased expression after SB216763 treatment. These overlapped with 1801 genes with high expression in ECs as compared with osteoblasts, and we identified another cohort of 235 genes that showed increased expression in both analyses. GO revealed these genes to be enriched in the signal pathway of endothelial differentiation and vascular formation (Supplemental Figure 3B).

We cultured the SB216763-treated osteoblasts in osteogenic induction media in vitro. Von Kossa staining showed a lack of mineralization (Supplemental Figure 3C). We then tested the SB216763-treated osteoblasts in tube formation assays in vitro, which showed a robust tube formation by VE-cadherin–positive cells (Supplemental Figure 3C). It suggested a suppression of osteoblastic characteristics after SB216763 treatment.

### SB216763 causes osteoblasts to lose osteogenic capacity in ectopic bone formation but gain ability to integrate into vascular endothelium structures.

**Figure 2:**
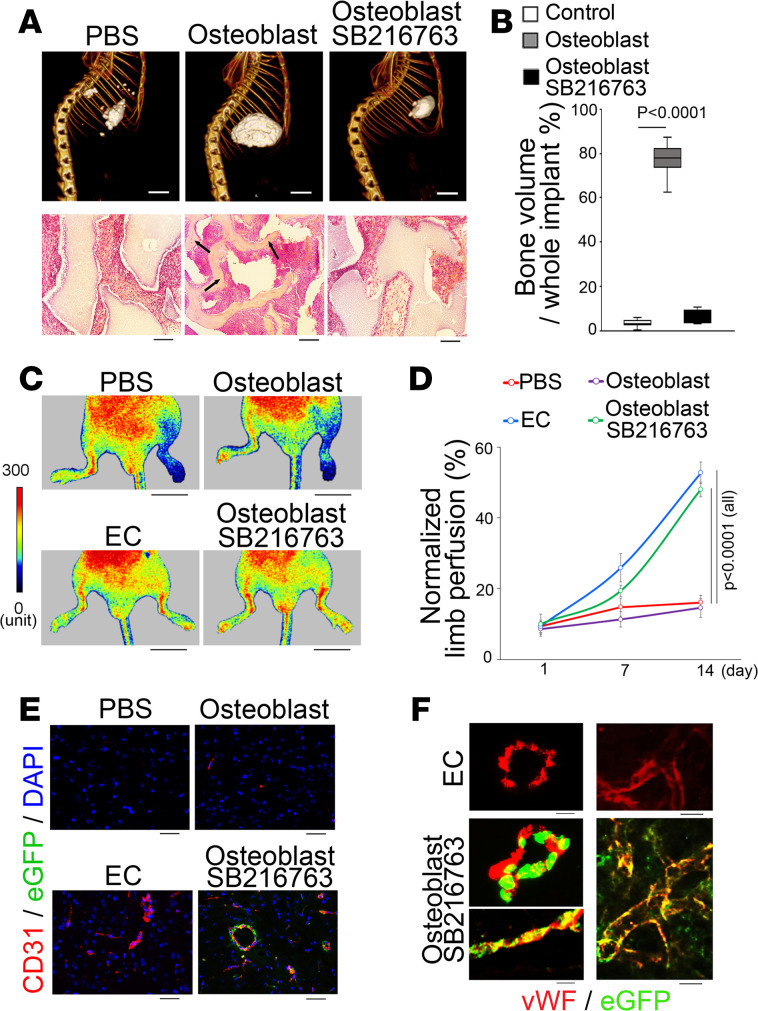
SB216763 treatment causes osteoblasts to lose osteogenic capacity in ectopic bone formation but gain ability to integrate into vascular endothelium and improve vascular repair. (**A**) Micro-CT images and H&E staining of ectopic bone formation after cell transplantation (*n* = 6). Black arrows indicate osteocytes. Scale bar: 5 mm (top); 50 μm (bottom). (**B**) Relative volume of bone formation (*n* = 6). (**C**) Laser Doppler perfusion images after cell transplantation (*n* = 8). Top, measurement camera. Bottom, documentation camera. ECs, mouse pulmonary endothelial cells. Scale bar: 10 mm. (**D**) Percentage of blood flow perfusion after cell transplantation normalized by perfusion of normal limb (*n* = 6). (**E**) Immunostaining of vessels after cell transplantation using anti-CD31 antibodies (*n* = 10). Scale bar: 50 μm. (**F**) EGFP-positive cells (green) were integrated into the endothelium of new vessels, which were stained with anti-vWF antibodies (red). Control, mouse pulmonary endothelial cells. PBS, phosphate-buffered saline with hydroxyapatite/tricalcium phosphate compound. Osteoblast, osteoblasts with hydroxyapatite/tricalcium phosphate compound. Osteoblast/SB216763, SB216763-treated osteoblasts with hydroxyapatite/tricalcium phosphate compound. **B** and **D** were analyzed for statistical significance by ANOVA with post hoc Tukey’s analysis. The bounds of the boxes are upper and lower quartiles. The line in the box is the median, and the whiskers are the maximum and minimal values. Data are shown as mean ± SD.

To assess the function of SB216763-treated osteoblasts, we performed transplantation experiments to examine the osteogenic capacity by using a well-established ectopic bone formation assay in vivo (22). We adapted the assay to the use of EGFP-positive cells, which were isolated from SB216763-treated Flk1-EGFP osteoblasts. Micro-CT imaging showed reduced amounts of ectopic bone, with less trabecular formation and bone volume in the implants containing the EGFP-positive cells compared with the controls (Figure 2, A and B). Histology and immunostaining both confirmed a lack of osteocytes in the implants with the EGFP-positive cells (Figure 2A and Supplemental Figure 4A).

Next, we tested the EGFP-positive cells in the hind limb ischemia model in nude mice to evaluate their capacity to promote vascular repair. After ligation of the proximal and distal femoral artery, EGFP-positive cells were transplanted. Mouse pulmonary ECs and osteoblasts were used as controls. Laser Doppler perfusion imaging demonstrated significantly higher limb blood flow in the mice transplanted with EGFP-positive cells than osteoblasts (Figure 2, C and D, and Supplemental Figure 4B), suggesting that the SB216763-treated cells gained endothelial function during the vascular repair. Histology and immunostaining also showed coexpression of EGFP and endothelial markers associated with the increased vascular density (Figure 2, E and F, and Supplemental Figure 4C), suggesting that the transplantation of the EGFP-positive cells enhanced vascularization through incorporation of additional cells or paracrine effects.

### Alteration of SMAD1 and β-catenin is responsible for osteoblastic-endothelial transdifferentiation.

**Figure 3:**
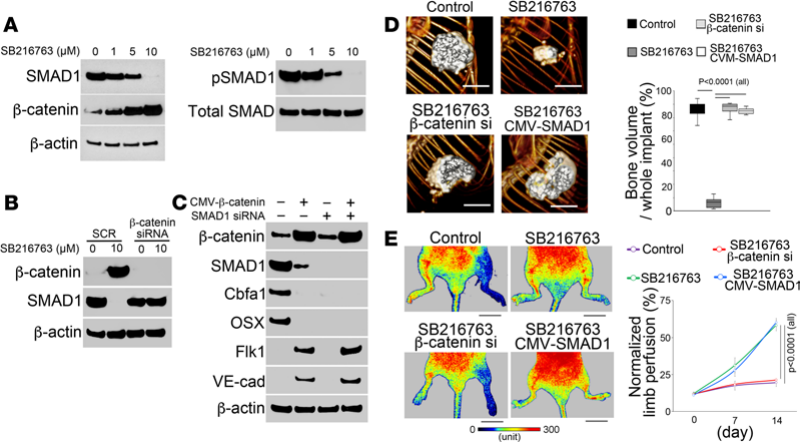
Enhanced β-catenin decreases SMAD1, and together they mediate SB216763-induced osteoblastic-endothelial transition. (**A**) Immunoblotting of SMAD1, phosphorylated SMAD1 (p-SMAD1), and β-catenin from osteoblasts treated with different doses of SB216763 (*n* = 6). (**B**) Immunoblotting of SMAD1 from osteoblasts transfected with β-catenin siRNA together with 10 μM SB216763 (*n* = 6). SCR, scrambled siRNA. (**C**) Immunoblotting of β-catenin, SMAD1, Cbfa1, osterix (OSX), Flk1, and VE-cadherin (VE-cad) from osteoblasts with overexpression of β-catenin or knockdown of SMAD1 or both (*n* = 6). β-Actin was used as control. CMV–β-catenin, CMV promoter–driven β-catenin cDNA. (**D**) Micro-CT images of ectopic bone formation and relative volume of bone formation in the implants after cell transplantation. Osteoblasts were used as controls. SB216763, SB216763-treated osteoblasts. SB216763/CMV-SMAD1, SB216763-treated osteoblasts infected with lentiviral vectors containing CMV promoter–driven SMAD1 cDNA. SB216763/β-catenin si, SB216763-treated osteoblasts infected with lentiviral vectors containing β-catenin siRNA (si) (*n* = 6). Scale bar: 5 mm. (**E**) Laser Doppler perfusion images and percentage of blood flow perfusion after cell transplantation (*n* = 8). Osteoblast and mouse pulmonary endothelial cells (ECs) were used as controls. Scale bar: 10 mm. **D** and **E** were analyzed for statistical significance by ANOVA with post hoc Tukey’s analysis. The bounds of the boxes are upper and lower quartiles. The line in the box is the median, and the whiskers are the maximum and minimal values. Data are shown as mean ± SD.

We screened the expression of transcription factors that are known to be involved in vascular development or bone formation. We found a dose-dependent decrease in expression and phosphorylation of SMAD1 but a robust increase of β-catenin in SB216763-treated osteoblasts as determined by immunoblotting (Figure 3A). GSK3-mediated phosphorylation of β-catenin is known to cause destabilization and degradation of the protein (23, 24), and the increase of β-catenin in the SB216763-treated osteoblasts validated these studies (Figure 3A). To determine whether excess β-catenin affected SMAD1 expression, we depleted β-catenin in SB216763-treated osteoblasts using siRNA. This prevented the decrease of SMAD1 (Figure 3B), suggesting that SMAD1 is a downstream target for suppression by β-catenin. We explored DNA-binding sites for β-catenin by locating binding motifs in the *Smad1* gene and identified 4 individual sites located at –196 bp, –1454 bp, –2805 bp, and –4729 bp upstream of exon1 (Supplemental Figure 5A). ChIP assays confirmed an increased abundance of β-catenin around these sites in the SB216763-treated osteoblasts compared with untreated controls (Supplemental Figure 5B). Next, we determined the transcriptional status of *Smad1* by examining the histone modification, including trimethylated histone H3 lysine 4 (H3K4me3), which is associated with active transcription, and H3K27me3, a marker of closed chromatin. The results showed decreased abundance of H3K4me3 but increased abundance of H3K27me3 around the same DNA-binding sites for β-catenin in the promoter region of the *Smad1* gene (Supplemental Figure 5C). Together, the results suggested that the increased β-catenin directly targeted the transcriptional regulation of *Smad1* and suppressed its expression.

We depleted β-catenin using lentiviral vectors containing β-catenin siRNA or restored SMAD1 using lentiviral vectors containing CMV promotor–driven SMAD1 cDNA in SB216763-treated osteoblasts (Supplemental Figure 5D). β-Catenin–specific siRNA efficiently reduced β-catenin and prevented the decrease of SMAD1. The restored SMAD1 had no effects on the β-catenin level (Supplemental Figure 5D), which supported β-catenin as an upstream regulator of SMAD1 expression.

To determine whether altering β-catenin and SMAD1 is sufficient for osteoblastic-endothelial transition, we first changed the levels of β-catenin and SMAD1 in osteoblasts using lentiviral vectors that expressed β-catenin or SMAD1 siRNA. We found that decreasing SMAD1 alone reduced expression of the osteogenic markers Cbfa1 and osterix but did not induce the endothelial markers Flk1 and VE-cadherin (Figure 3C). Increased β-catenin alone or in combination with decreased SMAD1 reduced the osteogenic markers and enhanced the endothelial markers (Figure 3C). The results suggested that the elevated β-catenin targeted SMAD1, diminished osteoblastic fate, and drove the cells toward the endothelial lineage.

We then assessed the osteogenic capacity of the SB216763-treated osteoblasts after restoring SMAD1 or depleting β-catenin. The cells were first cultured in osteogenic induction media. Von Kossa staining showed clear mineralization after restoring SMAD1 or depleting β-catenin in the SB216763-treated cells (Supplemental Figure 5E). We transplanted the cells into nude mice to test ectopic bone formation. Micro-CT imaging showed similar volumes of ectopic bone formation between osteoblast controls and SB216763-treated cells with SMAD1 overexpression or β-catenin depletion (Figure 3D). Immunostaining confirmed the presence of osteocytes in these implants (Supplemental Figure 5F). The results suggested that restoring SMAD1 or depleting β-catenin in SB216763-treated osteoblasts prevented the loss of osteogenic capacity.

Next, we tested the cells in tube formation assays in vitro. The results showed decreased tube formation only in SB216763-treated cells with β-catenin depletion (Supplemental Figure 5G). We transplanted the cells into the hind limb ischemia mouse model. Laser Doppler perfusion imaging showed less blood flow in the mice transplanted with SB216763-treated osteoblasts with depletion of β-catenin than cells without depletion (Figure 3E). No difference was observed between cells with or without restored SMAD1 (Figure 3E). The results suggested that depletion of β-catenin decreased the endothelial capacity of SB216763-induced osteoblasts in vascular repair.

We performed ChIP-Seq to examine the alterations of SMAD1 or β-catenin DNA binding in SB216763-treated osteoblasts. The Homer tool detected significant changes in the SMAD1 and β-catenin enrichment peaks (Supplemental Figure 6, A and B); 8214 genes were identified in which the DNA binding of SMAD1 decreased in the regulatory regions. We extended the search to determine whether there was any overlap between these genes and the 827 genes suppressed by SB216763 in osteoblasts. A new group of 649 genes was identified with a decrease in both gene expression and SMAD1 DNA binding. GO analysis termed these genes as being involved in the signaling pathways of osteoblastic differentiation and bone formation (Supplemental Figure 6A).

ChIP-Seq also showed 1543 genes with increased β-catenin DNA binding in the regulatory regions. Extended searches were conducted for potential overlaps between these genes and the cohort of 519 genes induced by SB216763 in the osteoblasts. A new group of 284 genes was identified with an increase in both expression and β-catenin DNA binding. GO analysis showed these genes to be involved in the signaling pathways of endothelial differentiation and vessel development (Supplemental Figure 6B). Collectively, the results revealed that SB216763 increased β-catenin to suppress SMAD1 and its transcriptional activity, which resulted in the loss of osteoblastic fate. On the other hand, an increase in β-catenin and its transcriptional activity led to endothelial differentiation.

### SB216763 prevents and shifts osteogenesis to reduce vascular calcification.

**Figure 4:**
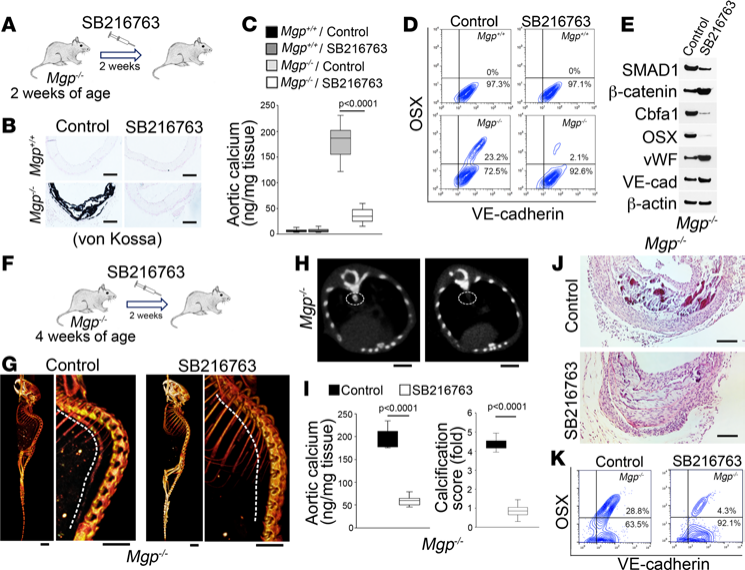
SB216763 promotes the transition of osteoblast-like cells to endothelial differentiation and ameliorates vascular calcification. (**A**) Schematic of the experimental procedure used to determine the effect of SB216763 treatment on early-stage calcification. (**B**) Von Kossa staining of aortic tissues (*n* = 7). Scale bar: 50 μm. (**C**) Total aortic calcium after SB216763 treatment (*n* = 8). (**D**) FACS analysis of CD31^+^ aortic cells using anti-osterix (OSX) and anti–VE-cadherin antibodies (*n* = 6). (**E**) Immunoblotting of aortic tissues of *Mgp^–/–^* mice after SB216763 treatment (*n* = 6). (**F**) Schematic of experimental procedures to examine the effect of SB216763 treatment on late-stage calcification. (**G** and **H**) Micro-CT images of aortic calcification in *Mgp^–/–^* mice after SB216763 treatment (*n* = 6). Scale bar: 5 mm. (**I**) Total aortic calcium and calcification score in *Mgp^–/–^* mice after SB216763 treatment (*n* = 8). (**J**) H&E staining of *Mgp^–/–^* aortic tissues after SB216763 treatment (*n* = 6). Scale bar: 50 µm. (**K**) FACS analysis of *Mgp^–/–^* aortic cells using anti-osterix (OSX) and anti–VE-cadherin antibodies (*n* = 6). Data in **C** were analyzed for statistical significance by ANOVA with post hoc Tukey’s analysis. Data in **I** were analyzed by unpaired Student’s *t* test. The bounds of the boxes are upper and lower quartiles. The line in the box is the median, and the whiskers are the maximum and minimal values.

To examine whether SB216763 could be used for treatment of vascular calcification, we chose the *Mgp^–/–^* mouse model, a well-known model of calcification (25). *Mgp^–/–^* mice develop arterial calcification as early as P14 (12), and at 4 weeks of age, all the arteries are severely calcified (25). We designed 2 independent experiments to test whether SB216763 decreased calcification in *Mgp^–/–^* mice. First, we treated young *Mgp^–/–^* mice at 2 weeks of age with SB216763 (5 μg/g daily) for 2 weeks to determine whether SB216763 prevented aortic osteogenesis (Figure 4A). After treatment, von Kossa staining showed less aortic mineralization in the SB216763-treated group than the controls (Figure 4B). The reduced calcium deposition in the SB216763-treated *Mgp^–/–^* mice was confirmed by quantification of total aortic calcium (Figure 4C). Aortic cell populations expressing CD31 were isolated and examined by FACS, which showed a decreased number of cells that coexpressed VE-cadherin and osterix but an increased number of cells that only expressed VE-cadherin in the SB216763-treated *Mgp^–/–^* mice (Figure 4D). Immunoblotting of aortic tissues further showed decreased Cbfa1 and osterix, increased vWF and VE-cadherin, and altered SMAD1 and β-catenin after SB216763 treatment (Figure 4E). The results suggested that SB216763 prevented vascular calcification by limiting EC-derived osteogenesis.

We then examined whether SB216763 was able to shift established osteogenesis in vascular calcification by treating *Mgp^–/–^* mice at 4 weeks of age for 2 weeks (Figure 4F). Micro-CT imaging showed a reduction in aortic calcification with a decrease in total aortic calcium and mineral deposition in the *Mgp^–/–^* mice after SB216763 treatment (Figure 4, G–I, and Supplemental Figure 7A). Furthermore, alizarin red staining of the entire aortas showed reduced mineralization in the SB216763-treated *Mgp^–/–^* mice (Supplemental Figure 7B). Histology results demonstrated a change of morphology in the calcified areas of the aortas (Figure 4J and Supplemental Figure 7C). FACS showed a decrease in aortic cells that coexpressed VE-cadherin and osterix, but an increase in cells that only expressed VE-cadherin after SB216763 treatment (Figure 4K). Again, real-time PCR showed decreased expression of osteogenic markers in the aortic tissue of the SB16763-treated *Mgp^–/–^* mice compared with untreated controls (Supplemental Figure 7D), suggesting that SB216763 reduced vascular calcification through osteoblastic-endothelial transition.

### Lineage tracing shows that SB216763 enhances the transition of osteoblast-like cells toward the endothelial lineage in vascular calcification.

**Figure 5:**
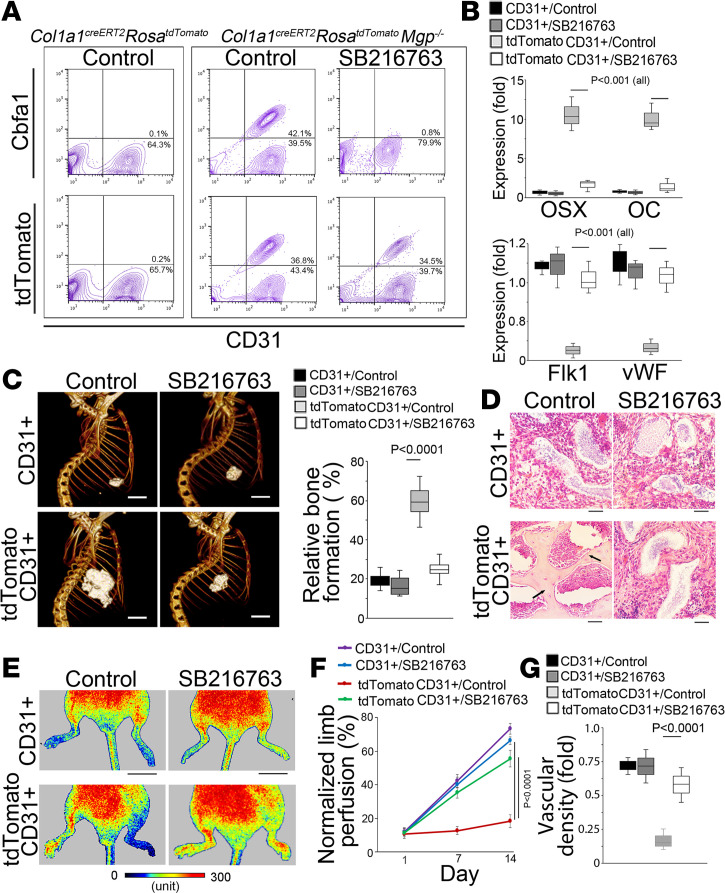
Osteoblastic lineage tracing reveals the transition of osteoblast-like cells to endothelial differentiation induced by SB216763 in calcified aortic tissue. (**A**) FACS analysis of aortic cells of *Col1α1^CreERT2^ Rosa^tdTomato^ Mgp^–/–^* mice treated with or without SB216763. (**B**) Expression of osterix (OSX), osteocalcin (OC), Flk1, and vWF in tdTomato^+^CD31^+^ cells isolated from aortic tissue of *Col1α1^CreERT2^ Rosa^tdTomato^ Mgp^–/–^* mice treated with or without SB216763 (*n* = 8). CD31^+^ cells were used as control. (**C**) Micro-CT images of ectopic bone formation and analysis of relative volume of bone formation after cell transplantation of CD31^+^ or tdTomato^+^CD31^+^ cells isolated from aortic tissue of *Col1α1^CreERT2^ Rosa^tdTomato^ Mgp^–/–^* mice treated with or without SB216763 (*n* = 6). Scale bar: 5 mm. (**D**) H&E staining of implants (*n* = 6). Black arrows indicate osteocytes. Scale bar: 50 μm. (**E**) Laser Doppler perfusion images after cell transplantation (*n* = 6). Scale bar: 10 mm. (**F**) Percentage of blood flow perfusion after cell transplantation normalized by perfusion of normal limb (*n* = 6). (**G**) Analysis of vascular density in ischemic sites after cell transplantation (*n* = 5). **B**, **C**, **F**, and **G** were analyzed for statistical significance by ANOVA with post hoc Tukey’s analysis. The bounds of the boxes are upper and lower quartiles. The line in the box is the median and the whiskers are the maximum and minimal values. Data are shown as mean ± SD.

Lineage tracing has previously suggested that osteoblast-like cells can be derived from ECs in calcified aortic tissue (12). These osteoblast-like cells express both osteogenic markers and the endothelial marker CD31 (12). To further investigate whether SB216763 would promote the transition of osteoblast-like cells toward endothelial differentiation in vascular calcification, we performed osteoblastic lineage tracing using *Col1α1^CreERT2^ Rosa^tdTomato^* mice, in which tamoxifen would translocate Cre to accomplish recombination and labeling of the osteoblasts with tdTomato (26–29). When the calcification started to develop on P14 (12), we treated the *Col1α1^CreERT2^ Rosa^tdTomato^ Mgp^–/–^* mice with tamoxifen (75 mg/kg, daily) for 5 consecutive days to label the osteoblast-like cells with tdTomato. At 4 weeks of age, FACS showed approximately 80% EC-derived osteoblast-like cells labeled with tdTomato after tamoxifen injection (Figure 5A). After 2 weeks of SB216763 treatment, 85% tdTomato-positive cells were still detected but without the osteogenic marker Cbfa1 (Figure 5A). We sorted and collected the tdTomato^+^CD31^+^ cells and found a decrease in osteogenic markers and a return of endothelial markers after SB216763 treatment (Figure 5B).

Again, we labeled the osteoblast-like cells with tdTomato in the aorta of the *Col1α1^CreERT2^ Rosa^tdTomato^ Mgp^–/–^* mice by tamoxifen injection and treated the mice with SB216763. We isolated tdTomato^+^CD31^+^ or CD31^+^ aortic cells and cultured them in osteogenic induction media. Von Kossa staining showed a lack of mineralization in the tdTomato^+^CD31^+^ cells of the SB216763 treatment group but extensive mineralization in the cells of the control group (Supplemental Figure 8A). In tube formation assays, however, the results showed strong tube formation by the tdTomato^+^CD31^+^ cells of the SB216763 treatment group but a lack of tube formation by the cells of the control group (Supplemental Figure 8B).

We used the same numbers of cells from each group to examine the osteogenic capacity in ectopic bone formation assays. Micro-CT imaging showed a smaller amount of ectopic bone with less trabecular formation and less bone volume in the implants with tdTomato^+^CD31^+^ cells from the SB216763 treatment group compared with controls (Figure 5C). Histology confirmed a lack of osteocytes in the implants with the SB216763 treatment cells (Figure 5D), suggesting that osteoblast-like cells lost their osteogenic capacity in the aortic tissues after SB216763 treatment.

We tested the capacity for vascular repair of the of tdTomato^+^CD31^+^ aortic cells using the hind limb ischemia model. Laser Doppler perfusion imaging showed much higher limb blood flow in the mice transplanted with the cells from the SB216763 treatment group than the controls (Figure 5, E and F, and Supplemental Figure 8C). Histology and immunostaining showed an increase in vascular density with integration of tdTomato-positive cells in the vascular endothelium from the same SB216763 treatment group (Figure 5G and Supplemental Figure 8D). It suggested that the osteoblast-like cells were directed toward the endothelial lineage by SB216763 in the aortic tissues.

### Limiting GSK3β recapitulates the transition of osteoblast-like cells to the endothelial lineage and reduces vascular calcification.

GSK3 has 2 isoforms, GSK3α and GSK3β (30). SB216763 specifically inhibits the activity of these GSK3 isoforms in an ATP-competitive manner (31). To determine which isoform of GSK3 was responsible for the osteoblastic-endothelial transition, we individually depleted GSK3α and GSK3β in mouse osteoblasts using specific siRNAs (Supplemental Figure 9A). The results showed a decrease in SMAD1 and osteogenic markers with an increase in β-catenin and endothelial markers only in the GSK3β-depleted osteoblasts (Supplemental Figure 9, A and B), suggesting that the inhibition of GSK3β drove osteoblastic cells toward endothelial differentiation.

**Figure 6:**
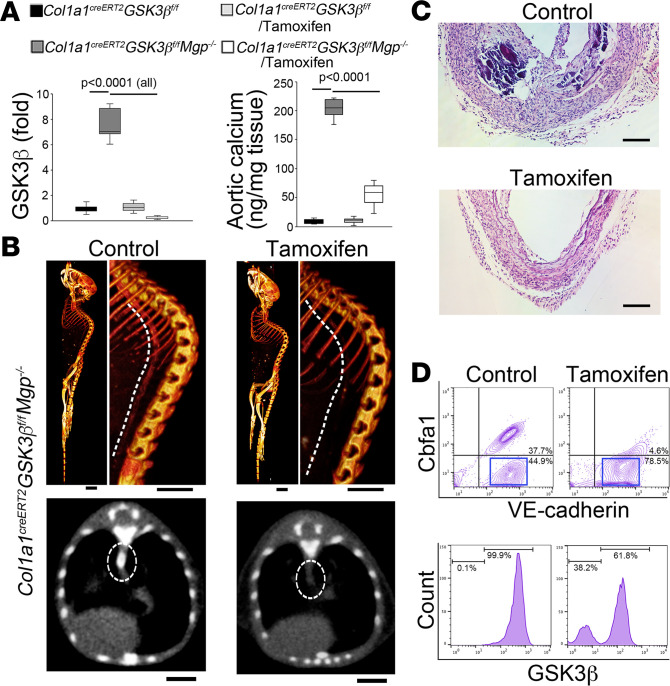
Specific gene deletion of GSK3β promotes osteoblast-like cells to endothelial transition and ameliorates vascular calcification. (**A**) Expression of GSK3β and total calcium in aortic tissues after tamoxifen injection (*n* = 8). (**B**) Micro-CT images and aortic calcification score of *Col1α1^creERT2^ GSK3β^fl/fl^ Mgp^–/–^* mice with or without injection of tamoxifen (*n* = 6). Scale bar: 5 mm. (**C**) H&E staining of *Col1α1^creERT2^ GSK3β^fl/fl^ Mgp^–/–^* aortic tissues with or without injection of tamoxifen (*n* = 8). Scale bar: 50 μm. (**D**) FACS analysis of aortic cells isolated from *Col1α1^creERT2^ GSK3β^fl/fl^ Mgp^–/–^* mice with or without injection of tamoxifen using anti-osterix (OSX) and anti–VE-cadherin antibodies (top) and VE-cadherin–positive cells (boxed) further examined using anti-*GSK3β* antibodies (bottom) (*n* = 6). **A** was analyzed for statistical significance by ANOVA with post hoc Tukey’s analysis. The bounds of the boxes are upper and lower quartiles. The line in the box is the median and the whiskers are the maximum and minimal values.

In lineage-tracing experiments using *Col1α1^CreERT2^* mice, we demonstrated that tamoxifen sufficiently translocated Cre to accomplish *loxp* recombination in osteoblast-like cells in *Mgp^–/–^* aortic tissues. To determine the effect of GSK3β gene deletion on osteoblast-like cells, we generated *Col1α1^CreERT2^ GSK3β^fl/fl^ Mgp^–/–^* mice. At 4 weeks of age when the aortas were severely calcified, we injected the mice with tamoxifen for 5 days. Ten days after injection, we confirmed the reduction of GSK3β in aortic tissues (Figure 6A). Total aortic calcium deposition and micro-CT imaging showed a decrease of aortic calcification in the *Col1α1^CreERT2^ GSK3β^fl/fl^ Mgp^–/–^* mice after deletion of GSK3β (Figure 6, A and B, and Supplemental Figure 9C). Histology confirmed that a change in morphology had occurred in the osteoblast-like cells (Figure 6C and Supplemental Figure 9D). FACS revealed an increase in ECs without expression of osteogenic markers in the aortic tissue of the tamoxifen-treated group. In addition, the increased endothelial population contained a subgroup of cells in which GSK3β expression was abolished (Figure 6D). Real-time PCR further showed that the deletion of GSK3β decreased osteogenic markers in the aortic tissues (Supplemental Figure 9E), suggesting that the deletion of GSK3β allowed the transition in osteoblast-like cells and reduced the calcification.

### SB216763 treatment does not affect bone formation.

To determine whether long-term treatment of SB216763 affects bone formation, we treated WT mice at 8 weeks of age with SB216763 (5 μg/g daily) for 8 weeks. The experiment was designed to test the effect on normal bone. *Mgp^–/–^* mice were excluded from this test because of the abnormalities already reported in *Mgp^–/–^* bones (25). After treatment, the bones were examined by micro-CT imaging and immunostaining. Micro-CT imaging showed no differences in relative bone volumes or connectivity densities between SB216763-treated and untreated controls (Supplemental Figure 10, A and B). Immunostaining for CD31 and osterix showed no change in the microstructure of the bone tissues or the vasculature (Supplemental Figure 10C). In addition, no hemorrhages were detected in the bone tissue (Supplemental Figure 10C). We examined the GSK3β expression in the tdTomato-labeled osteoblasts of normal bone and osteoblast-like cells of calcified aorta. We found that GSK3β expression was more than 25-fold higher in the aortic osteoblast-like cells than the bone osteoblasts (Supplemental Figure 10D). Specific gene deletion further showed a more robust decrease of GSK3β in the calcified aortic tissue than the normal bone (Supplemental Figure 10E), suggesting that the mature osteoblasts in the bone with low levels of GSK3β had a limited response to SB216763 treatment.

## Discussion

In this study, we found that the inhibition of GSK3β allowed transition, full or partial, from osteoblastic to endothelial lineage by modulating β-catenin and SMAD1 and that this transition improved vascular calcification. The results provide evidence for osteoblastic-endothelial transition as a potentially novel concept and revealed a previously unknown role of GSK3β in balancing these lineages. The newly identified compound SB216763 may be a candidate for treatment of vascular calcification.

In observational studies, vascular calcification is diagnosed in more than 60% of the population above 65 years of age, including calcification of the internal elastic lamina, media, atherosclerotic lesions, and more extreme forms such as coral reef aorta and porcelain aorta (32). The calcification is a severe complication that increases all-cause mortality from cardiovascular disease, diabetes mellitus, and chronic kidney disease but lacks medical treatment. Here, we discovered a process that redirects osteoblast-like cells in calcified lesions that could be considered in treatment strategies aimed at various types of vascular calcification. The strategy may also limit fibrodysplasia ossificans progressive (FOP), a rare disease in which ECs contribute to osteogenesis in fibrous tissues (1).

Transition of ECs to osteoblast-like cells has been well documented in recent studies. It has been shown that enhanced BMP signaling triggers endothelial-mesenchymal transitions, which cause the ECs to gain plasticity and transition into osteoblast-like cells. These can subsequently contribute to vascular calcification in *Mgp* deficiency, diabetes mellitus, and atherosclerosis (1, 12, 14, 16, 18, 19, 33). Studies have further shown that excess BMP activity induces a group of specific serine proteases in the ECs that promote the endothelial-mesenchymal transitions and assist the EC-derived osteoblast-like cells in migrating into the aortic wall (13, 14, 20). Osteoblast-like cells derived from ECs are also found in calcific processes related to FOP and cancer (1, 18). In the current study, lineage tracing showed that labeled ECs expressed osteoblastic markers or that labeled osteoblast-like cells expressed endothelial markers in the calcified vessels, indicating that the ECs undergo transitions into osteoblast-like cells in vascular calcification. In this study, we found that GSK3β inhibition had the potential to transition the osteoblast-like cells back to endothelial differentiation. Although the results showed that SB216763 switched the profile of osteoblastic differentiation close to endothelial differentiation and rendered EC-derived osteoblast-like cells able to regain endothelial function, the gene expression profile did not show the exact match, suggesting the possibility of incomplete transition of these cell fates. This may leave us a further challenge to investigate how to guide the ill-fated ECs totally back to normalization.

Small molecules have a number of advantages and have been used as valuable tools for modulating or directing cell differentiation (34, 35). Small molecules can directly modify proteins or DNA to change cell phenotypes. In well-designed screens, specific small molecules have been found to manipulate stem cells to differentiate into multiple lineages such as cardiomyocytes (36), neurons (37), and hematopoietic stem cells (38). Treatment with small molecules can also induce pluripotency or transdifferentiation in mature cells (39, 40). They are commonly used for studying mechanisms and may be appropriate for clinical translation. We combined high-throughput screens with a lineage reporter in creating a potentially novel approach that identifies small molecules that induce lineage transitions. This type of screening would provide candidates able to modulate cell differentiation in cardiovascular disease and accelerate the identification of small molecules for translational research.

GSK3 is a serine/threonine kinase with constitutive activation in unstimulated cells (30). The activity of GSK3 is regulated by serine phosphorylation in response to extracellular signals (41) and plays different roles in osteogenic and endothelial differentiation. GSK3 promotes osteogenic differentiation (42), and GSK3 deficiency disrupts the maturation of osteoblasts resulting in a reduction of bone formation (43). In contrast, GSK3 prevents endothelial differentiation, and inhibition of GSK3 promotes the differentiation, proliferation, and migration of ECs (44, 45). We showed that the inhibitory effect of SB216763 on GSK3β allowed osteoblast-like cells to transition to endothelial differentiation and reduced vascular calcification. Other GSK3 inhibitors include lithium chloride (LiCl), maleimide derivatives (indolyl-maleimide inhibitors, 3-anilino-4-arylmaleimides1-3, orbisindolyl maleimide and benzofuranyl-indolyl maleimide inhibitors), staurosporine and organometallic inhibitors, indole derivatives, paullone derivatives, pyrazolamide derivatives, pyrimidine and furopyrimidine derivatives, oxadiazole derivatives, and thiazole derivatives. With the knowledge obtained from this study, it may be worth exploring the effect of these inhibitors on vascular calcification.

The SMADs constitute a family of transcription factors with 8 members, SMAD1–8 (46). After activation by TGF-β or BMP signals, phosphorylated SMADs are translocated to the nuclei to regulate the transcription of target genes (47). The level of SMAD1 is essential for osteoblastic differentiation because it is a critical mediator of BMP signals (48). Increased SMAD1 activity promotes osteoblastic differentiation, whereas a decrease reduces osteoblastic differentiation in osteoprogenitor cells (49, 50). SMAD1 protein levels are regulated by GSK3 activity in sensory axon regeneration (51). We found that the inhibition of GSK3β decreased the ability of SMAD1 to direct osteoblastic differentiation through β-catenin induction.

β-Catenin is a member of the catenin protein family and is expressed in many tissues. It is a mediator of the canonical Wnt signal pathway, which is essential for endothelial differentiation (24). β-Catenin also interacts with Notch to regulate endothelial specification (45). Because GSK3-mediated phosphorylation of β-catenin causes destabilization and degradation, the activity of GSK3 is critical for β-catenin levels (23, 24). We found that GSK3 inhibition increased β-catenin and suppressed SMAD1, which in turn changed their transcriptional activities and led to the transition of osteoblasts to endothelial differentiation. The results not only revealed how β-catenin and SMAD1 balanced the transcriptional landscapes of endothelial and osteogenic differentiation but also indicated that the activators of β-catenin or the inhibitors of SMAD1 affect vascular calcification. This may include methyl vanillate or Wnt agonist 1 for β-catenin activation and myricetin for SMAD1 inhibition.

Interestingly, excess β-catenin reduced SMAD1 and suppressed osteoblastic differentiation in osteoblasts while inducing endothelial differentiation. The depletion of SMAD1 alone, however, was unable to induce the endothelial differentiation, suggesting that β-catenin targets earlier drivers of endothelial differentiation than those targeted by SMAD1. Indeed, β-catenin has been shown to directly target early genes in endothelial differentiation, such as Sox17 and delta-like ligand 4 (Dll4) (24, 52). Also, β-catenin interacts with recombination signal binding protein for immunoglobulin kappa J region (RBPJ) and Notch intracellular domains to regulate endothelial differentiation (45). Further studies will be required to identify if excess β-catenin targets these pathways in the activation of endothelial transitions in osteoblasts.

Altogether, this study identified a method for treating or preventing vascular calcification by administering the GSK3β inhibitor SB216763, which redirected osteogenesis to endothelial differentiation through induction of β-catenin and suppression of SMAD1.

## Methods

### Animals.

*Mgp^+/–^*(B6.129S7-Mgptm1Kry/KbosJ), *Col1α1^CreERT2^* [B6.Cg-Tg(Col1α1-Cre/ERT2)1Crm/J], and *GSK3β^fl/fl^* [B6.129(Cg)-Gsk3btm2Jrw/J] on a C57BL/6J background were purchased from The Jackson Laboratory. Genotypes were confirmed by PCR, and experiments were performed with generations F4–F6. Littermates were used as WT controls. All mice were fed a standard chow diet (Harlan Teklad Laboratory, 8604).

### Tissue culture.

The osteoblast cell line MC3T3 was purchased from American Type Culture Collection (CRL-2593) and cultured as per the manufacturer’s protocol. SB216763 (MilliporeSigma, S3442) treatment was performed as described in the main text. Lentiviral vectors containing CMV-SMAD1, SMAD1 siRNA, CMV–β-catenin, or β-catenin siRNA were all purchased from GeneCopoeia and applied to the cells as per the manufacturer’s protocols.

### Micro-CT imaging.

Micro-CT imaging was performed at the Crump Institute for Molecular Imaging at UCLA. All the samples were scanned on a high-resolution, volumetric micro-CT scanner (μCT125). The image data were acquired with the following parameters: 10 μm isotropic voxel resolution; 200 ms exposure time; 2000 views; and 5 frames per view. The micro-CT–generated DICOM files were used to analyze the samples and to create volume renderings of the regions of interest. The raw data files were viewed using the MicroView 3D volume viewer and analysis tool (GE Healthcare) and AltaViewer software. Additionally, images of the samples were generated using SCIRun (Scientific Computing and Imaging Institute).

### Laser Doppler perfusion imaging.

Laser Doppler perfusion imaging was performed using a real-time microcirculation imaging system (Perimed). The imaging was conducted under normal ambient room lighting. A 20 × 27 mm high-resolution model was used with a 1388 × 1038 pixel measurement camera and a 752 × 580 pixel documentation camera at 1 image per second. The image resolution was set to 20 μm/pixel and 21 images per frame until stopped. Windows-based PIMSoft software (Perimed) was used to process the data.

### Mouse surgery.

All the surgeries were performed on a heated pad with a connection to a continuous flow of isoflurane. Ectopic bone formation was performed as previously described (22): 5 × 10^5^ cells were mixed with 40 mg hydroxyapatite/tricalcium phosphate powder (Salvin Orastruct 0.5 cc) and incubated in a 1 mL syringe at 37°C in 5% CO_2_ overnight. After disinfection with 70% ethanol, a skin incision was made on the back of the mouse. A subcutaneous pouch was formed by blunt dissection. The mixture of cells and hydroxyapatite/tricalcium phosphate was transplanted into the pouch and the incision was closed. The implants were examined by micro-CT imaging and histology 12 weeks after transplantation.

The murine model of hind limb ischemia was performed as previously described (53). A 10 mm long incision of the skin was made toward the medial thigh. The femoral artery was exposed and separated from the femoral vein and nerve. Silk sutures were used to tie the proximal and distal ends of the femoral artery with double knots. The cells (5 × 10^5^) were transplanted into the surgical area and the incision was closed. Laser Doppler perfusion imaging was used to monitor the blood flow at different time points. Histology and immunostaining were used to examine the vascularization 2 weeks after transplantation.

### RNA analysis.

Real-time PCR analysis was performed as previously described (14). GAPDH was used as a control gene (14). Primers and probes for mouse and human Cbfa1, osterix, osteocalcin, CD34, VE-cadherin, CD31, eNOS, SMAD1, and β-catenin were obtained from Applied Biosystems (Thermo Fisher Scientific) as part of TaqMan gene expression assays.

### Tube formation assays.

Tube formation assays were performed as previously described (54). Briefly, Matrigel (BD Biosciences) was diluted 1:3 in medium, and 300 μL was added to each well of a 12-well plate and incubated at 37°C for 30 minutes to allow polymerization. Cells were suspended in the same medium at a density of 5 × 10^4^ cells/well, and 400 μL of the cell suspension was added to each well. Photographs were obtained after 6 hours using a Nikon Eclipse TE2000-S microscope with a Nikon Plan Fluor 4× objective lens. The images were captured using an INFINITY2 camera (Lumenera) and analyzed by ImageJ (NIH).

### FACS.

FACS analysis was performed as previously described (14). The cells were stained with FITC- or phycoerythrin-conjugated antibodies against CD31 and VE-cadherin (all from BD Biosciences, 553372 and 562243) and osterix (Santa Cruz Biotechnology, sc-22536). Nonspecific fluorochrome- and isotype-matched IgGs (BD Pharmingen, 563636 and 554121) served as controls.

### Immunoblotting and immunofluorescence.

Immunoblotting was performed as previously described (12). Equal amounts of tissue lysates were used for immunoblotting. Blots were incubated with specific antibodies against SMAD1, GSK3α, and GSK3β (all from Cell Signaling Technology, 9743, 433T, and 93115); β-catenin and Cbfa1 (all from R&D Systems, AF1329 and MAB2006); osterix (Santa Cruz Biotechnology, sc-22536); Flk1 and VE-cadherin (all from BD Biosciences, 55307 and 562242); and vWF (Dako, A0082). β-Actin (MilliporeSigma, A2228) was used as a loading control. Immunofluorescence was performed as previously described in detail (12). We used specific antibodies against CD31 (BD Biosciences, 553370), osterix (Santa Cruz Biotechnology, sc-22536), and vWF (Dako, A0082). The nuclei were stained with DAPI (MilliporeSigma, D9564).

### RNA-Seq, ChIP-Seq, and ChIP assays.

For RNA-Seq, osteoblasts were treated with 10 μM SB216763 for 14 days, and mouse pulmonary ECs were used as controls. Total RNA was isolated using RNeasy Mini Kit (QIAGEN). The libraries were constructed and the sequencing was conducted using the Illumina HiSeq 3000 system by the Technology Center for Genomics & Bioinformatics at UCLA. Spliced Transcripts Alignment to Reference (STAR) was used for the read alignment and gene counts. We used R package limma and edgeR to perform differential gene expression, GO analysis, and pathway enrichment analysis. Log_2_ fold change of 2 and FDR of 0.01 were used as cutoffs to identify differentially expressed genes. RNA-Seq data were deposited in the NCBI’s Gene Expression Omnibus (GEO; GSE167962).

For ChIP-Seq, specific antibodies were used to enrich the genomic DNA as described (55). ChIP DNA was sequenced by the Technology Center for Genomics & Bioinformatics at UCLA. Reads from each sample were mapped to the mouse genome (mm10) using Bowtie2. The Homer tool was used to detect significant enrichment of peaks with FDR 0.05 and more than 4-fold over input. Motif occurrences in peaks were identified by the Homer motif discovery function. Peak annotation was performed to associate peaks with nearby genes and calculate tag densities. GO analysis and pathway enrichment of the identified genes were also performed. We used specific antibodies for SMAD1 (Cell Signaling Technology, 9743) and β-catenin (R&D Systems, MAB2006). The data were deposited in the NCBI’s GEO (GSE147374).

ChIP assays were performed as previously described (55). We used specific antibodies for β-catenin (R&D Systems, MAB2006), H3k4me3 (Abcam, ab8580), and H3k27me3 (Abcam, ab6002). The primers for the real-time PCR were as follows: 5′-GAAAATAACACAGGCTTTG-3′ and 5′-GCTCCCCGAGCCTGGATT-3′; 5′-GGACAGAGGCTCTCATTCC-3′ and 5′-CAATTCTTGGATCTCATCTTA-3′; 5′-GGGTGACCAAGCATGCTAGC-3′ and 5′-CCTGGCCACCTCCATCTTGC-3′; 5′-GGAGAGGCCATGTTGAGGAC-3′ and 5′CCTAGCGTCTACACTGGGTAG-3′.

### High-throughput system for the compound screen.

MC3T3 cells were stably infected with Flk1 promoter–driven EGFP by using Flk1-EGFP lentivirus (GeneCopoeia). The plates were coated with laminin (20 μg/mL) and washed with PBS twice using an ELx405 plate washer (Bio-Tek Instruments). Cells in 25 μL medium per well were loaded by Multidrop 384 (Thermo Fisher Scientific), and the chemical compounds were pinned to the plates with media. GFP-positive cells (positive controls) and WT cells (negative controls) were also seeded. The plates were transferred to an STX220 CO_2_ plate incubator (LiCONiC Instruments) and incubated. The plates were transferred and delivered by a Thermo Fisher Scientific Spinnaker robot. EGFP expression was determined and imaged using a FlexStation II and Victor 3V (PerkinElmer) every day for 2 weeks.

### Statistics.

The analyses were performed using GraphPad InStat, version 3.0. Data were analyzed by either unpaired 2-tailed Student’s *t* test or 1-way ANOVA with Tukey’s multiple-comparison test for statistical significance. *P* values less than 0.05 were considered significant.

### Study approval.

The studies were reviewed and approved by the IRB and conducted in accordance with the animal care guidelines set by UCLA. The investigation conformed to the *Guide for the Care and Use of Laboratory Animals* (National Academies Press, 2011).

## Author contributions

YY supervised the experiments, analyzed data, and wrote the manuscript. JY, XW, XQ, DZ, LZ, JM, XC, and KIB performed experiments and data analysis.

## Supplementary Material

Supplemental data

## Version 1 04/13/2021

In-Press Preview

## Version 2 05/24/2021

Electronic publication
