# Supplementary material for: Shifting osteogenesis in vascular calcification
Source: JCI Insight. 2021 May 24;6(10):e143023. doi: 10.1172/jci.insight.143023 (PMC8262274; doi:10.1172/jci.insight.143023)
Supplement: Supplemental data [file jciinsight-6-143023-s106.pdf]

Supplemental Information

Shifting Osteogenesis in Vascular Calcification

Jiayi Yao, M.D., Ph.D.[1], Xiuju Wu, M.D., Ph.D.[1], Xiaojing Qiao Ph.D.[1], Daoqin Zhang Ph.D.[1], Li Zhang Ph.D.[1], Jocelyn Ma, B.S.[1], Xinjiang Cai, M.D., Ph.D.[1], Kristina I. Boström, M.D., Ph.D.[1, 2], and Yucheng Yao, M.D., Ph.D.[1, *]

* Correspondence: Yucheng Yao, M.D., Ph.D.

This PDF file includes:

Supplemental Figures S1 to S10

Supplemental Figure Legends S1 to S10

Supplemental Figure and Legends

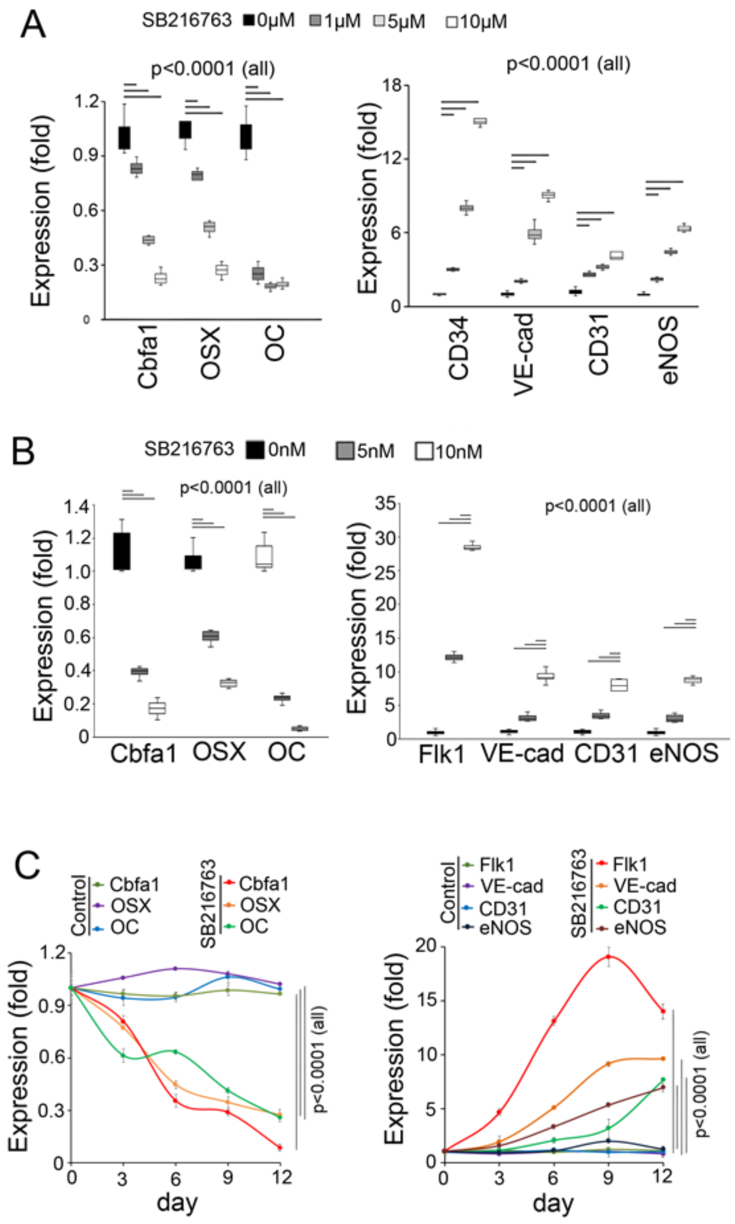

Figure 1. ***SB216763 to drive osteoblasts to endothelial differentiation.*** **(A)** Expression of the osteogenic markers Cbfa1, osterix (OSX), osteocalcin (OC) and the endothelial markers CD31, VE-cadherin, CD34, eNOS in osteoblasts treated with different does of SB216763 (n=8). **(B)** Expression of the osteogenic markers Cbfa1, osterix (OSX), osteocalcin (OC) and the endothelial markers CD31, VE-cadherin, CD34, eNOS in human osteoblasts treated with different doses of SB216763 (n=8). **(C)** Time-course expression of the osteogenic markers Cbfa1, osterix (OSX), osteocalcin (OC) and the endothelial markers CD34, VE-cadherin, CD31, eNOS in SB216763-treated human osteoblasts (n=8). Data were analyzed for statistical significance by ANOVA with post hoc Tukey's analysis. The bounds of the boxes are upper and lower quartiles. The line in the box is median and the whiskers are the maximum and minimal values. Error bars are mean ± standard deviation (SD).

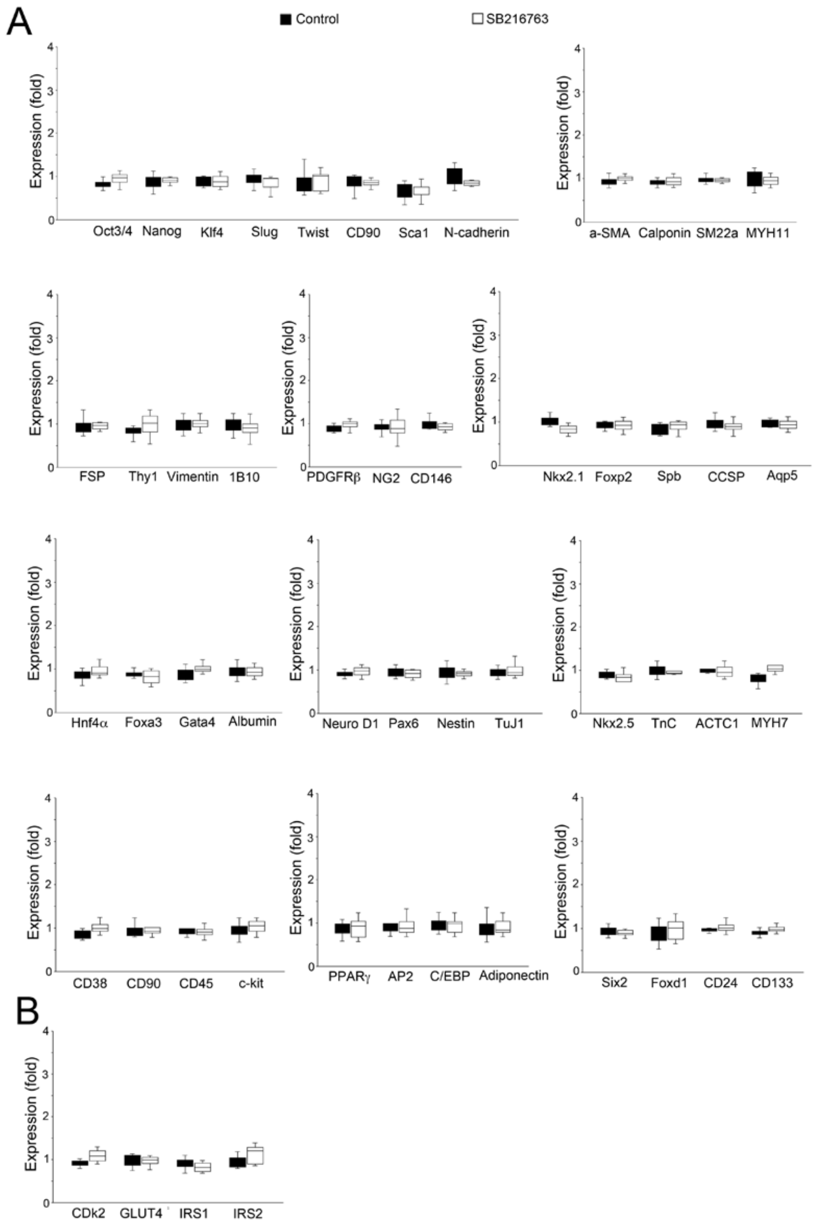

Figure 2. ***SB216763 to drive osteoblasts to endothelial differentiation*.** **(A)** Expression of markers for different lineages examined by real-time PCR. The lineages included mesenchymal and stem cell, smooth muscle cell, fibroblast, pericyte, adipocyte, pulmonary, hepatic, neuronal, cardiac, hematopoietic and renal lineages (n=6). α-SMA, alpha smooth muscle actin. MYH11, myosin heavy chain 11. FSP, fibroblast-specific protein. NG2, neuron-glial antigen 2. Spb, surfactant protein b. CCSP, club-cell secretory protein. Aqp5, aquaporin 5. Tnc, troponin c. ACTC1, actin alpha cardiac muscle 1. MYH7, myosin heavy chain 7. AP2, adipocyte protein 2. C/EBP, CCAAT/ enhancer binding protein. **(B)** Expression of representative genes in glucose metabolism (n=6). CK2, casein kinase 2. GLUT4, glucose transporter 4. IRS1 and 2, insulin receptor substrates 1 and 2. Data were analyzed for statistical significance by ANOVA with post hoc Tukey's analysis. The bounds of the boxes are upper and lower quartiles. The line in the box is median and the whiskers are the maximum and minimal values.

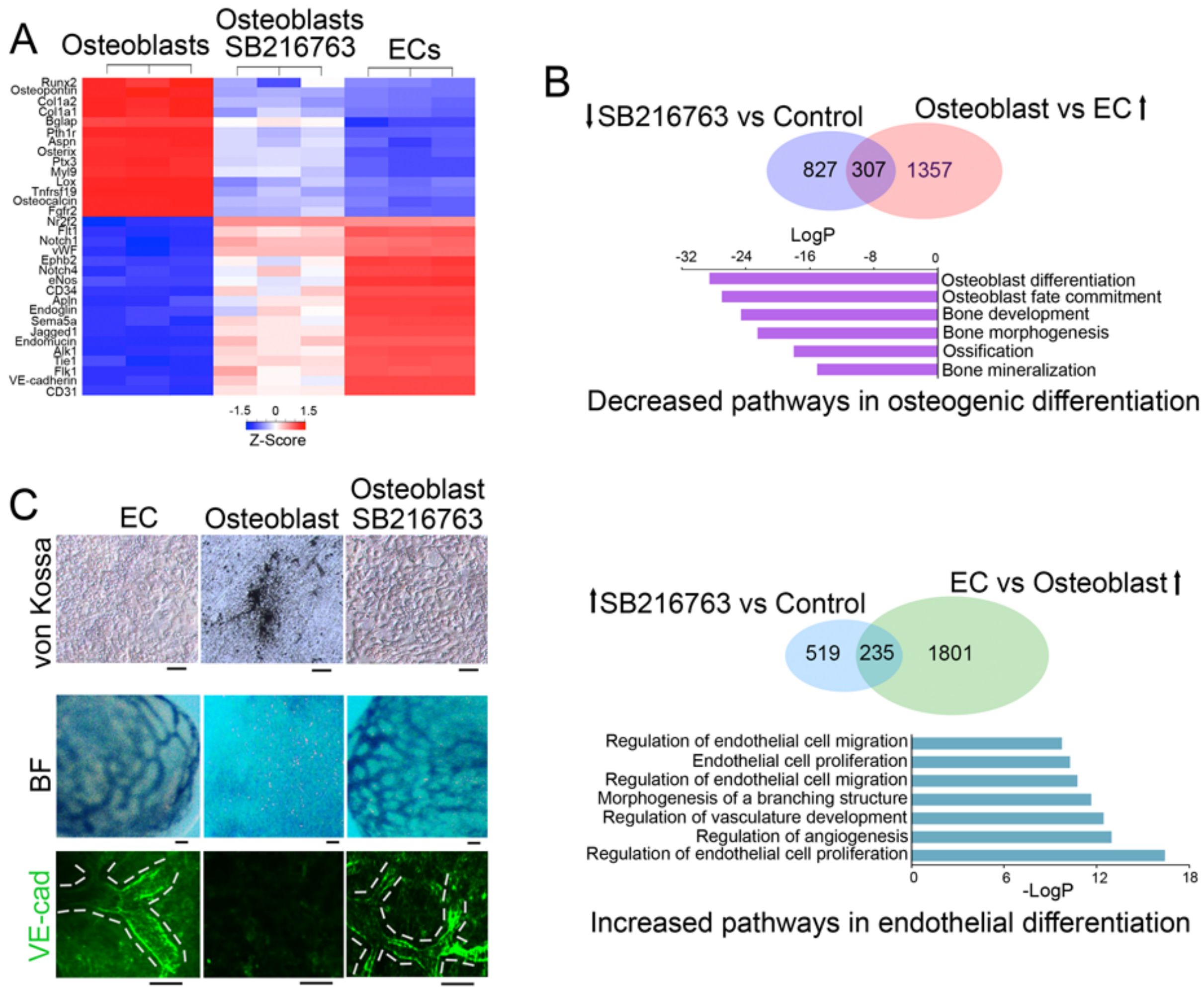

Figure 3. ***SB216763 to drive osteoblasts to endothelial differentiation*.** **(A)** A heatmap of expression profile of SB21673-treated osteoblasts versus controls (n=3). Mouse pulmonary endothelial cells (EC) were isolated by FACS and used as controls. **(B)** Top, GO analysis of a cohort of 307 genes with decreased expression in SB216763-treated osteoblasts and high expression when comparing osteoblasts to endothelial cells. Bottom, GO analysis of a cohort of 235 genes with both increased expression in SB216763-treated osteoblasts and high expression when comparing endothelial cells to osteoblasts. Log 2-fold change of 2 and FDR 0.01 were used as differential cut-off. **(C)** Osteogenesis assay of SB216763-treated osteoblasts stained by von Kossa (top) (n=5). Tube formation assay of SB216763-treated osteoblasts (middle) and stained by anti-VE-cadherin (VE-cad) antibodies (bottom) (n=5). Scale bar, 50 μm. BF, bright field. EC, mouse pulmonary endothelial cells.

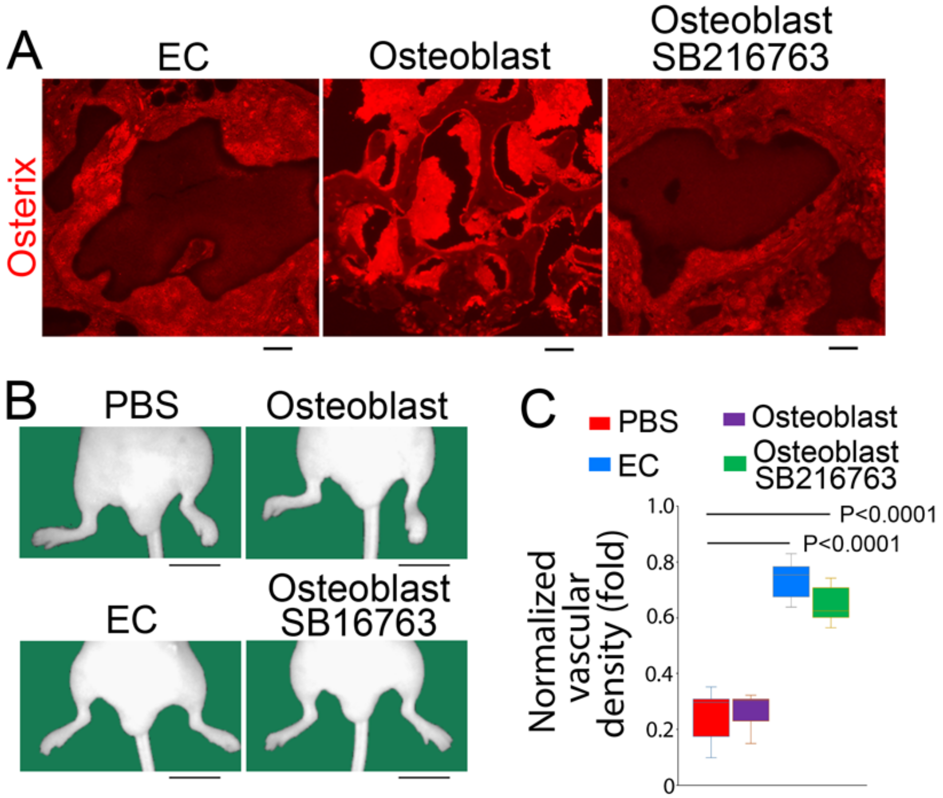

Figure 4. ***SB216763 treatment causes osteoblasts to lose osteogenic capacity in ectopic bone formation but gain ability to integrate into vascular endothelium to improve vascular repair*.** **(A)** Immunostaining of the sections of implants of SB216763-treated osteoblasts with anti-osterix antibodies (n=6). Scale bar, 50 µm. **(B)** Laser Doppler perfusion images with documentation camera after cell transplantation (n=8). PBS, phosphate buffered saline. EC, mouse pulmonary endothelial cells. Scare bar, 10 mm. **(C)** Analysis of vascular density in ischemic sites after cell transplantation (n=10). C was analyzed for statistical significance by ANOVA with post hoc Tukey's analysis. The bounds of the boxes are upper and lower quartiles. The line in the box is median and the whiskers are the maximum and minimal values.

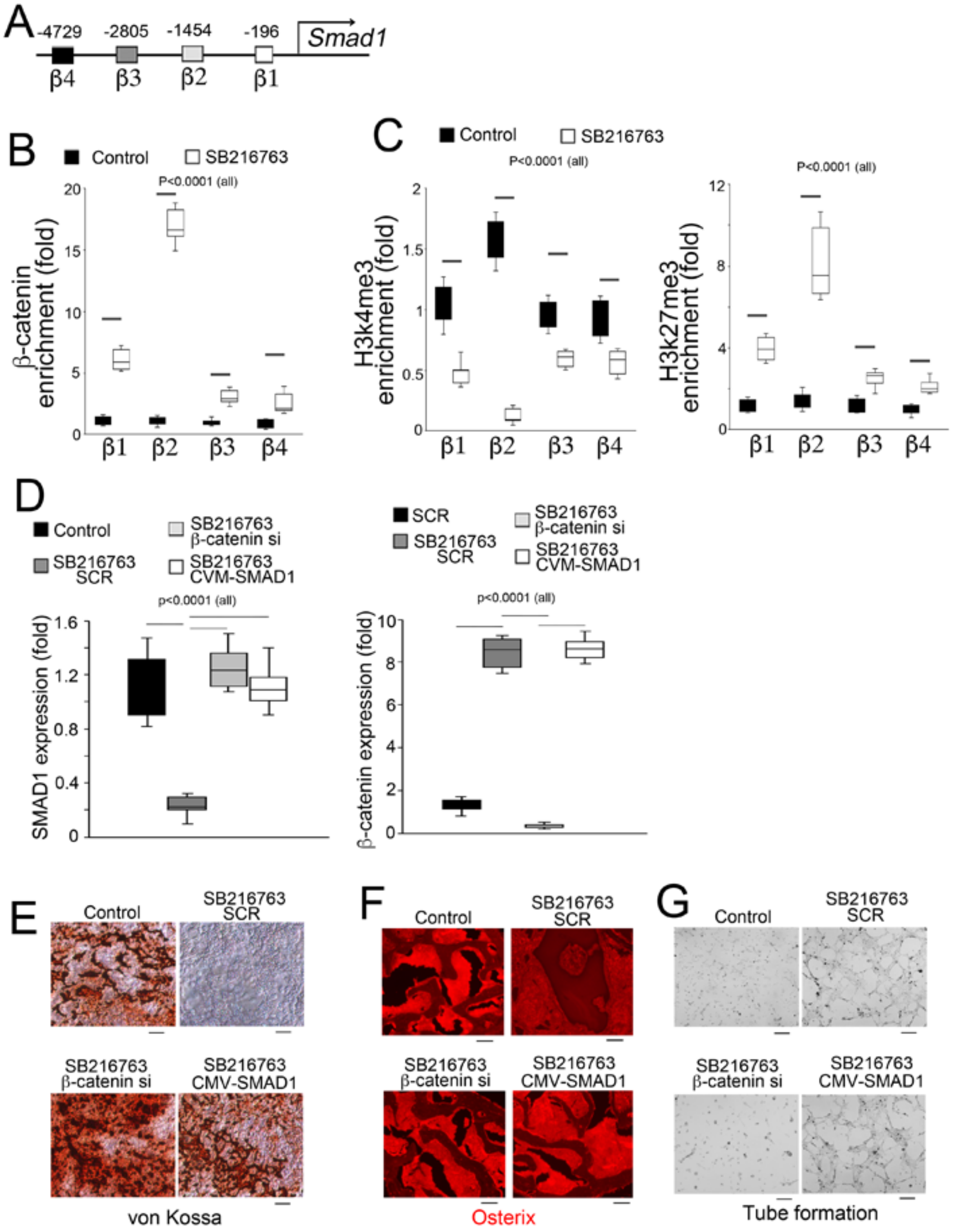

Figure 5. *Increased β-catenin decreases SMAD1 and together are responsible for SB216763 to induce osteoblastic-endothelial transdifferentiation*. (A) DNA-binding sites of β-catenin in promoter region of Smad1 gene. **(B-C)** ChIP assay of DNA-binding of β-catenin and histone modification in promoter of Smad1 gene. **(D)** Expression of SMAD1 or β-catenin in SB216763-treated osteoblasts infected with lentiviral vectors containing CMV promoter-driven SMAD1 cDNA or lentiviral vectors containing β-catenin siRNA (n=6). **(E)** Osteogenesis assay stained with alizarin red (n=6). Scale bar, 50 μm. **(F)** Immunostaining of sections of implants with anti-osterix antibodies (n=6). Scale bar, 50 μm. **(G)** Tube formation assay (n=6). B, C and D were analyzed for statistical significance by ANOVA with post hoc Tukey's analysis. The bounds of the boxes are upper and lower quartiles. The line in the box is median and the whiskers are the maximum and minimal values.

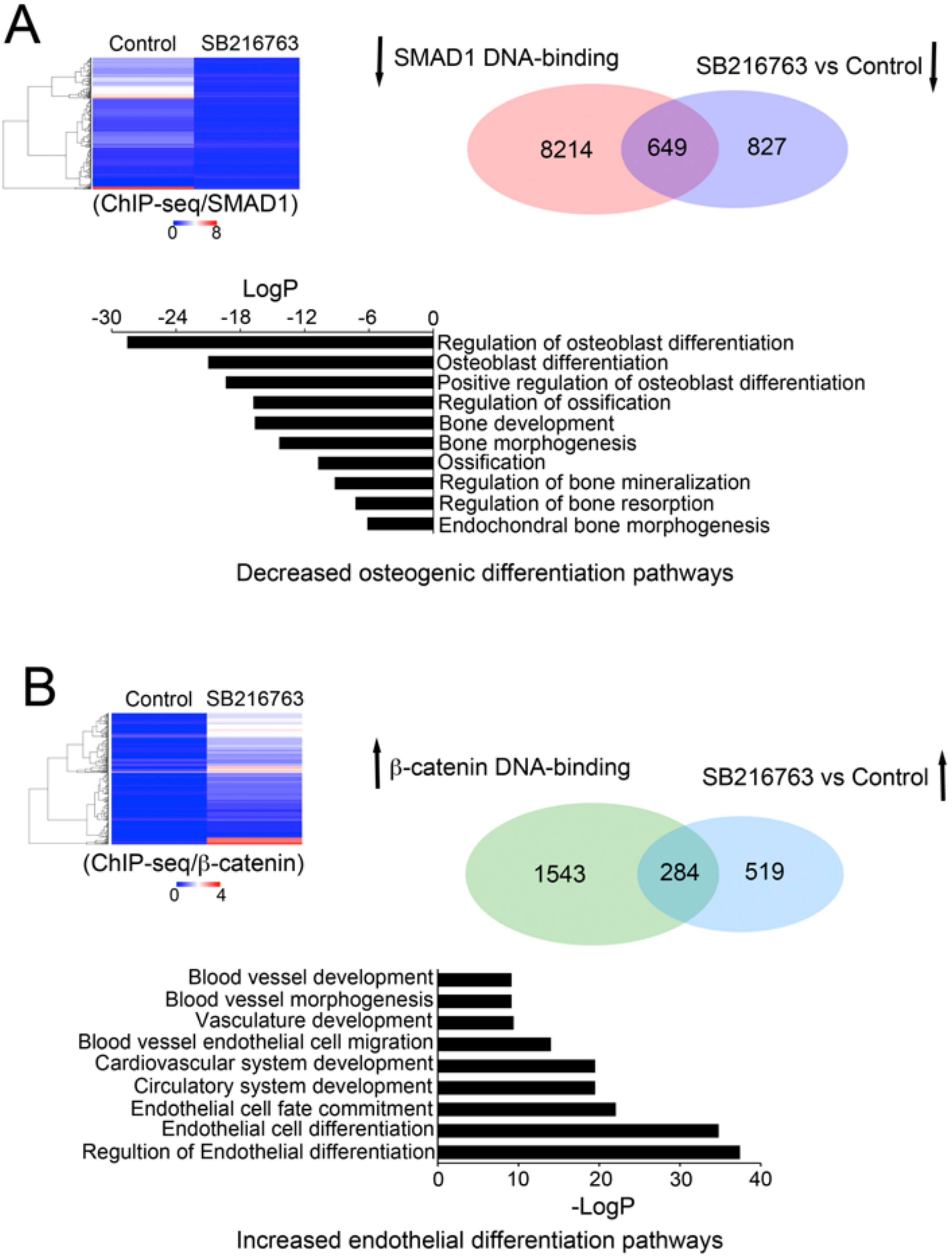

Figure 6. *Modulation of β-catenin and SMAD1 alter transcriptional landscapes of osteoblasts.* (A) Heatmap of the cohorts of genes with decreased SMAD1 DNA-binding and GO analysis of the genes with decreased SMAD1 DNA-binding and decreased expression in SB21673-treated osteoblasts. **(B)** Heatmap of the cohorts of genes with increased β-catenin DNA-binding in SB21673-treated osteoblasts and GO analysis of the genes with increased β-catenin DNA-binding and increased expression in SB21673-treated osteoblasts.

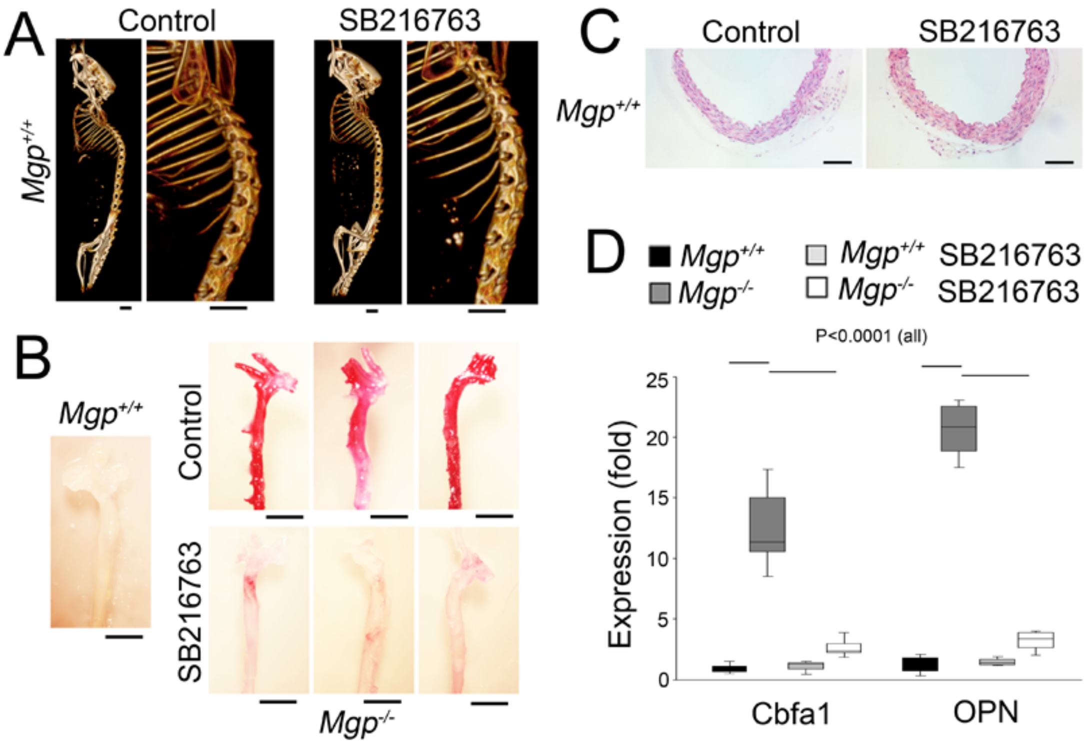

Figure 7. ***SB216763 shifts osteogenesis to endothelial differentiation to ameliorate vascular calcification in Mgp$^{-/-}$ mice*.** **(A)** Micro-CT images of wild type (*Mgp*$^{+/+}$) mice after SB216763 treatment (n=6). Scale bar, 5 mm. **(B)** Alizarin red staining of aortic tissues (n=6). Scale bar, 5 mm. **(C)** H&E staining of aortic tissues of *Mgp*$^{+/+}$ mice after SB216763 treatment (n=6). Scale bar, 50 µm. **(D)** Expression of cbfa1 and osteopontin (OPN) in aortic tissues of *Mgp*$^{-/-}$ and *Mgp*$^{+/+}$ mice after SB216763 treatment (n=8). D was analyzed for statistical significance by ANOVA with post hoc Tukey's analysis. The bounds of the boxes are upper and lower quartiles. The line in the box is median and the whiskers are the maximum and minimal values.

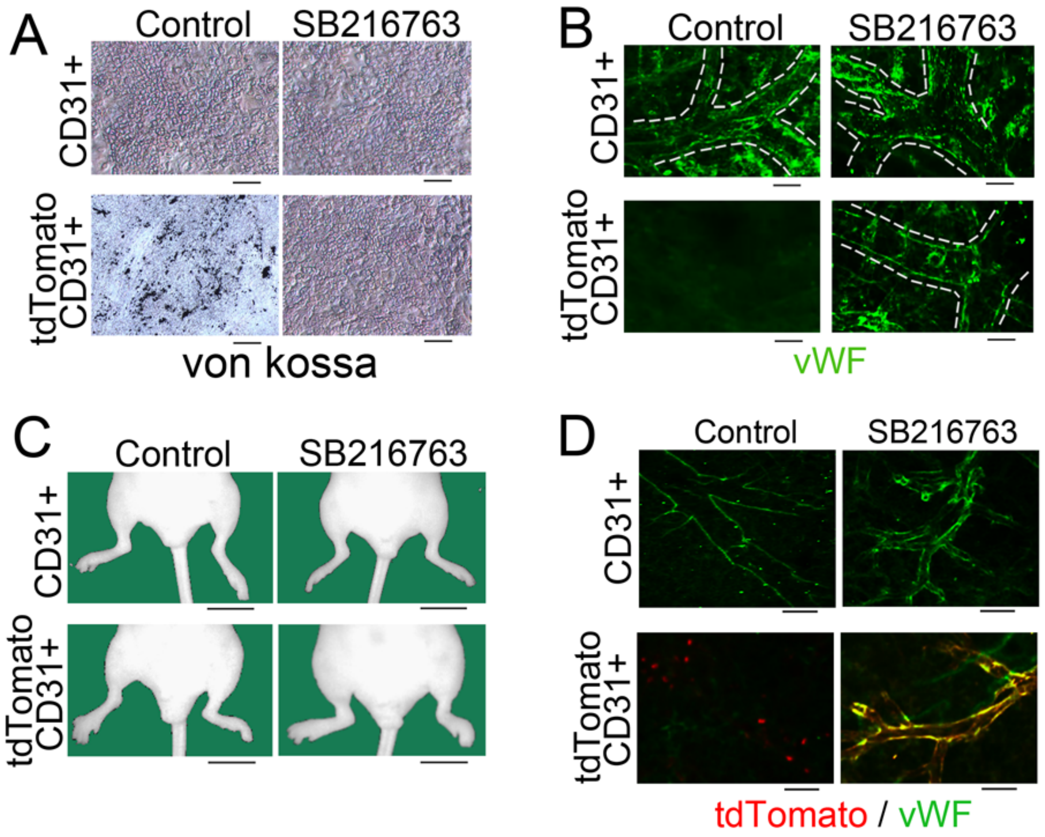

Figure 8. ***Osteoblastic lineage-tracing reveals the shift of osteoblast-like cells to endothelial differentiation by SB216763 in calcified aortic tissue***. **(A)** Von Kossa staining of osteogenesis assay and **(B)** vWF staining of tube formation assay by using tdTomatoCD31+ cells isolated from aortic tissue of $Col1\alpha1^{CreERT2}Rosa^{tdTomato}Mgp^{-/-}$ mice treated with or without SB216763 (n=6). CD31+ cells were used as control. Scale bar, 50 μm. **(C)** Laser Doppler perfusion images with documentation camera after cell transplantation (n=6). Scare bar, 10 mm. **(D)** Immunostaining by using anti-vWF antibodies in ischemic sites after cell transplantation (n=5).

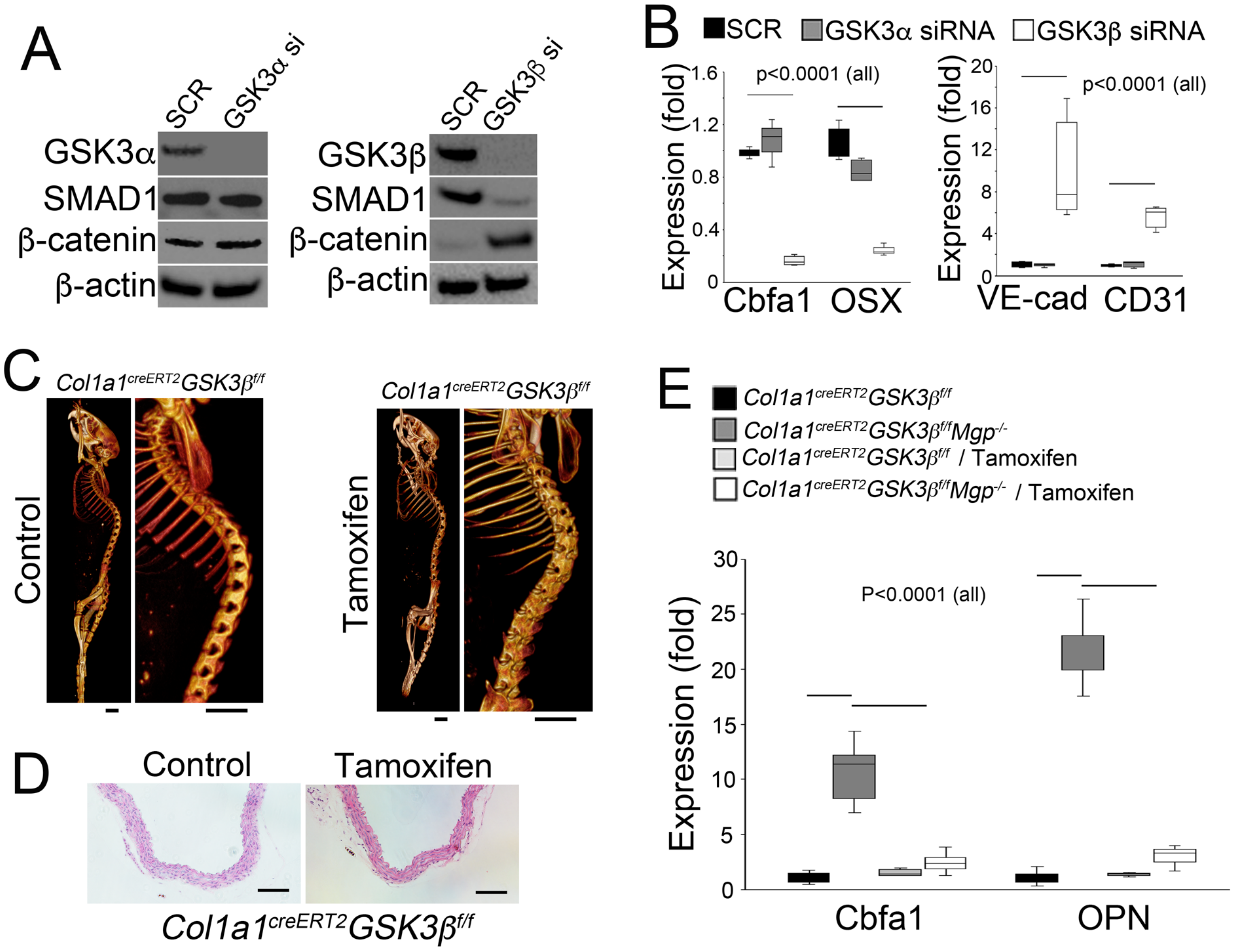

Figure 9. ***Specific deletion of GSK3β reduces vascular calcification.*** **(A)** Immunoblotting of osteoblasts transfected with GSK3α or GSK3β siRNA (n=6). β–actin was used as loading control. **(B)** Real-time PCR showing the expression of Cbfa1, osterix (OSX), VE-cadherin (VE-cad) and CD31 in osteoblasts transfected with GSK3α or GSK3β siRNA (n=6). SCR, scramble siRNA. **(C)** Micro-CT images of mice after injection of tamoxifen (n=6). Scale bar, 5 mm. **(D)** H&E staining of aortic tissues of mice after injection of tamoxifen (n=8). Scale bar, 5 mm. **(E)** Expression of Cbfa1 and osteopontin (OPN) in aortic tissues of mice after injection of tamoxifen (n=8). B and E were analyzed for statistical significance by ANOVA with post hoc Tukey's analysis. The bounds of the boxes are upper and lower quartiles. The line in the box is median and the whiskers are the maximum and minimal values.

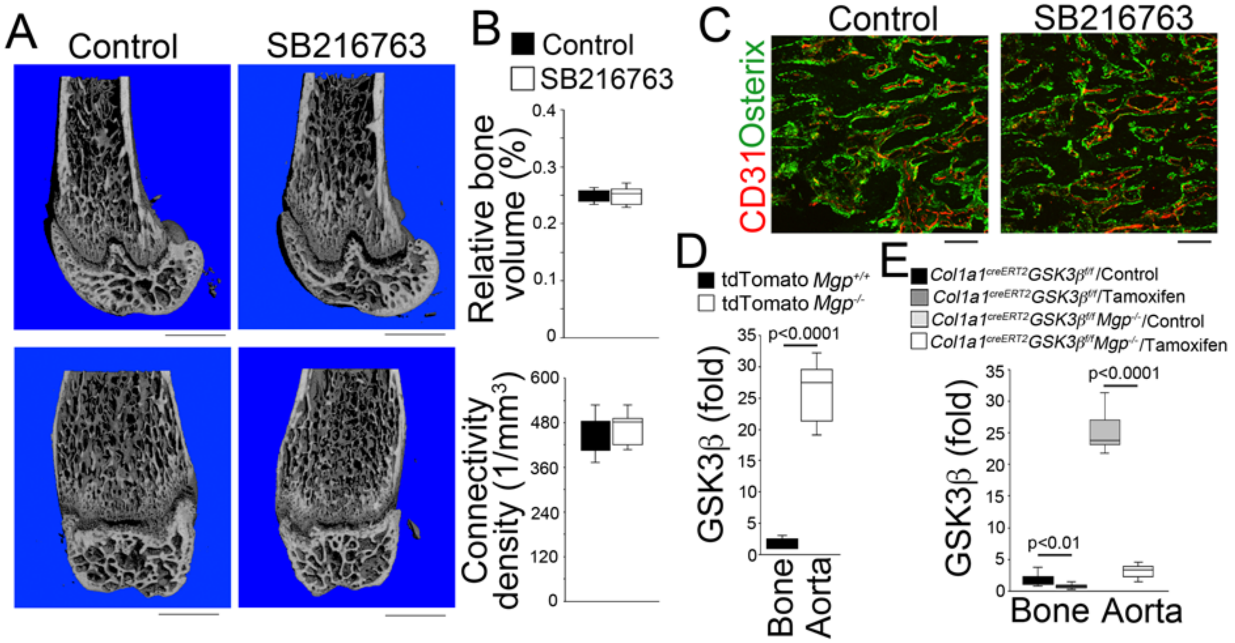

Figure 10. ***SB216763 treatment has not shown impact on bone formation*****.** **(A)** Micro-CT imaging of bone tissues of wild type mouse treated with or without SB216763 (n=6). Scale bar, 1mm. **(B)** Relative bone volume and connectivity density of bone tissues of wild type mice treated with or without SB216763 (n=6). **(C)** Immunostaining of bone tissues of wild type mice by using anti-CD31 or anti-osterix antibodies (n=6). Scale bar, 50µm. **(D)** *GSK3β* expression in the tdTomato-positive cells from bone of $Col1\alpha1^{CreERT2}Rosa^{tdTomato}$ mice and aorta of $Col1\alpha1^{CreERT2}Rosa^{tdTomato}Mgp^{-/-}$ mice after tamoxifen injection (n=7). **(E)** *GSK3β* expression in the bone of $Col1\alpha1^{CreERT2}GSK3\beta^{flox/flox}$ and the aorta of $Col1\alpha1^{CreERT2}GSK3\beta^{flox/flox}Mgp^{-/-}$ mice after tamoxifen injection (n=5). B and D were analyzed by unpaired Student's t test. E were analyzed for statistical significance by ANOVA with post hoc Tukey's analysis. The bounds of the boxes are upper and lower quartiles. The line in the box is median and the whiskers are the maximum and minimal values.
